# Open STM: A low-cost scanning tunneling microscope with a fast approach method

**DOI:** 10.1016/j.ohx.2023.e00504

**Published:** 2023-12-21

**Authors:** Weilin Ma

**Affiliations:** Institute of Optics and Electronics, Chinese Academy of Sciences, Chengdu 610209, China; University of Chinese Academy of Sciences, Beijing 100049, China

**Keywords:** Open-source hardware, HOPG imaging, Educational instrument, Piezoelectric slider, Approach algorithm

## Abstract

In this study, we have developed an low-cost scanning tunneling microscope (STM) cost of 300 USD or 2000 CNY. The microscope is suitable for educational purposes and low-demand research imaging at the nanometer level. The microscope's motion components and scanner are controlled using piezoelectric materials, avoiding the thermal drift associated with traditional motor control. Our tip approach algorithm, which considers the capacitance and friction characteristics during piezoelectric slider movement, has reduced the time required for sample loading to establish tunneling current to approximately 1 min. The physical dimensions of the microscope body are 45 × 45 × 31.5 mm (W × L × H), and the control voltage does not exceed 15 V, ensuring the safety of operators, particularly those with limited experience. During performance verification, we conducted a scanning tunneling scan on a Highly Oriented Pyrolytic Graphite (HOPG) sample, utilizing bias voltages of 50 mV and 60 mV, resulting in clear observations of the atomic features of HOPG within the STM pattern.

## Graphical abstract

**Figure:**
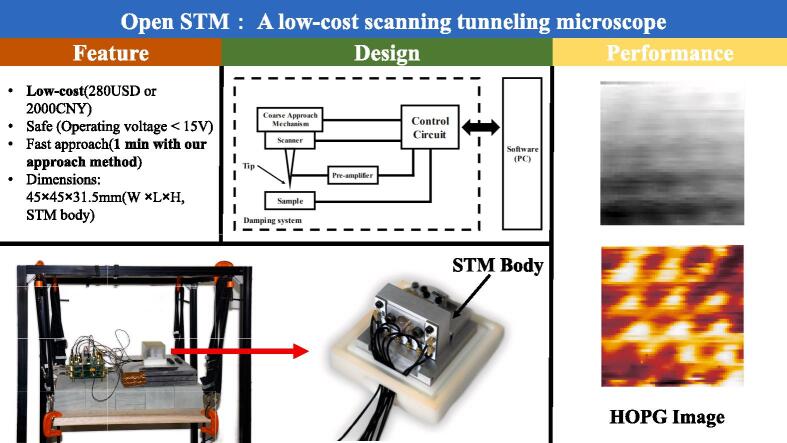

Specifications table

**Table:** 

Hardware name	Open STM(Open-sources scanning tunneling microscope)
Subject area	• Engineering and materials science • Educational tools and open source alternatives to existing infrastructure
Hardware type	Imaging tools
Closest commercial analog	No commercial analog is available.
Open source license	CC-BY-SA-4.0 and GPLv3(Software, / Python files) License
Cost of hardware	300USD or 2000CNY
Source file repository	https://doi.org/10.17632/f35c6xzzcm.1

## Hardware in context

Since the advent of the Scanning Tunneling Microscope (STM) in 1981, pioneered by Binnig and Rohrer of IBM, who were subsequently awarded the Nobel Prize in 1986 [1], [2], this instrument has become widely employed in scientific research due to its exceptional capabilities for sub-nanometer scale imaging. The STM enables the visualization of individual atoms and molecules across diverse materials, facilitating a profound understanding of their spatial arrangement, organizational structures, and inherent defects. However, its utilization is predominantly confined to academic and research contexts, as the technical expertise required for its operation renders it inaccessible to the general public. Typically, universities and research institutions acquire the STM through scientific instrument vendors at a cost ranging from $8,000 (for low-end models) to $30,000-$150,000 (for professional-grade instruments) [3].

In 1986, Besocke introduced an easily operable STM design[4] for an STM that does not necessitate a vacuum or low-temperature environment. This design incorporates a tripod structure constructed with piezoelectric tubes to facilitate STM imaging. While the piezoelectric tube employed remains relatively expensive, the foundational principles of simplicity and stability delineated in Besocke's work have become pivotal in subsequent designs. In approximately 2001, Alexander presented a straightforward STM design on his website, enabling the construction of a low-cost STM through do-it-yourself (DIY) methods [5]. Alexander innovatively devised a novel scanner, termed the “unimorph disk scanner,” based on his patent [6]. This design involves segmenting a piezoelectric buzzer into four parts, costing approximately $1, and applying voltage to these sections individually to attain three-dimensional control. The introduction of this scanner significantly mitigated overall costs. In 2015, Berard documented the creation of a home-built STM achieving atomic resolution [7]. Based on the design specifications provided, replication of the STM is feasible with reasonable cost and complexity. Berard adopted Alexander's scanner design and implemented a tripod lifting structure with a stepper motor to execute the coarse approach procedure. However, the open-loop nature of this structure precludes the estimation of the distance between the sample and the tip. Consequently, the coarse approach procedures necessitate considerable time to prevent tip collision. Additionally, thermal expansion resulting from the stepper motor's heating introduces instability in the spatial relationship between the sample and the tip. In 2022, Liao et al. introduced a low-cost nanopositioner leveraging the stick–slip effect to achieve nanometer-level movement [8]. Notably, the positioning slider of this apparatus, utilizing piezoelectric ceramics as actuators, circumvents heat generation. Integrating this positioner as a coarse approach mechanism for the STM addresses approach speed and thermal drift concerns.

**Fig. 1:**
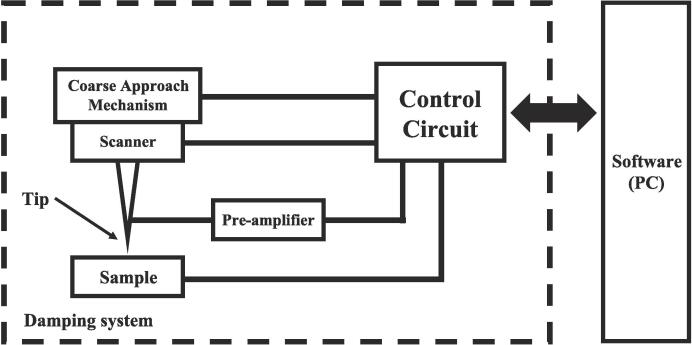
Schematic of an STM.

In the STM imaging process, a bias voltage is applied to the sample, bringing it and the tip into a tunneling distance typically ranging from 4 to 7 Å [9]. A piezoelectric component controls the tip for scanning and imaging, and the resulting tunneling current reflects changes in the sample's surface morphology. The essential components of the STM design, as depicted in Fig. 1, include:

• **Scanner:** The scanner enables controlled movement in three axes(XYZ) of the tip, facilitating translational scanning of the sample surface.
• **Coarse Approach Mechanism:** Responsible for controlling the movement of the sample towards the tip, ensuring its placement within the scanning range of the scanner.
• **Vibration Isolation System:** Maintaining a stable distance between the tip and the sample during scanning is crucial for accurate measurements. Two effective designs are commonly employed for STM systems: spring suspension vibration isolation or a vibration isolation platform composed of Viton and stacked metal plate[10], [11], [12]. Magnetic damping can further enhance vibration isolation in the suspension system.
• **Pre-amplifier:** Given that tunneling currents typically range from picoamps to nanoamps [13], a pre-amplifier is essential for detecting such minuscule currents using conventional circuits. Common designs include Transimpedance Amplifiers (TIA) and instrumentation amplifiers [14].
• **Control Circuit:** The control circuit controls the various components of the STM system and facilitates communication with a computer. This includes the transmission of commands, signals, and other pertinent information.
• **Software:** Operational software is indispensable for controlling the STM and facilitating image processing and storage. This software plays a vital role in operating and utilizing the microscope system.

Drawing inspiration from the contributions of Alexander, Berard, and Liao et al., we have developed a low-cost STM cost of $300 or 2000 CNY. This microscope employs comprehensive piezoelectric control, effectively mitigating issues associated with thermal drift. Additionally, we have identified an electrical signal during the coarse approach process that serves as a reference for the tip-sample distance, significantly reducing the time required to establish tunneling current from sample loading (decrease to 1 min).

## Hardware description

**Fig. 2:**
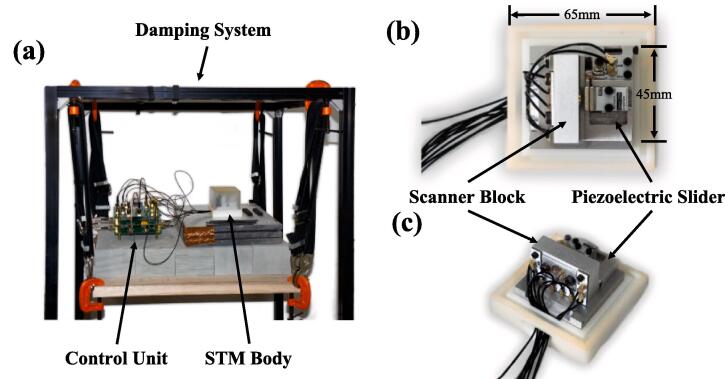
The assembled STM. (a) The microscope body and control unit are placed on a simple damping system. (b) Top view of the STM body with the dust cover open. (c) Side view of the STM body.

**Fig. 3:**
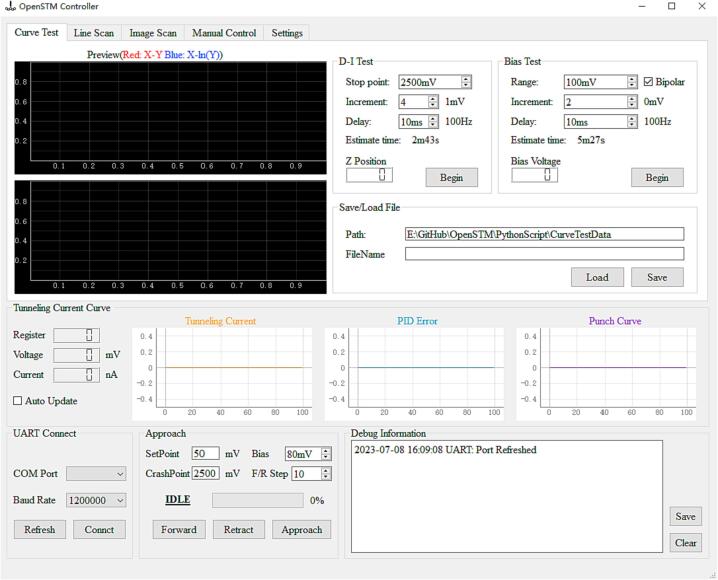
PC software interface.

This paper introduces an easy-to-operate, cost-effective STM design capable of attaining atomic-level imaging on Highly Oriented Pyrolytic Graphite (HOPG) surfaces. The microscope configuration encompasses a control unit, an STM body, and a vibration-damping system, as delineated in Fig. 2(a). The dimensions of the microscope body are specified as 65 × 65 × 55 mm (W × L × H), while the core unit, excluding the dust cover, measures 45 × 45 × 31.5 mm (W × L × H), as presented in Fig. 2 (b). Motion control of the STM is facilitated through piezoelectric materials integrated with our proprietary control algorithms. This innovation diminishes the coarse approach time to 1 min, obviating the necessity for external optical microscopes and capacitance detection circuits [15]. Moreover, the system operates with a maximum voltage not exceeding 15 V, thus mitigating the risk of electrical shock during operation and ensuring the safety of less experienced operators. As depicted in Fig. 3, our software provides fundamental imaging capabilities and incorporates diverse testing functionalities, such as tunneling distance-current curve testing, bias voltage testing, and scanner repeatability testing. Test outcomes are promptly displayed and stored in real time.

The STM developed herein demonstrates applicability in educational and research domains (low demand).

• In the educational sphere, this microscope can serve as an instructive tool for explaining the tunneling principle in quantum mechanics. Its inherent ease of replication makes it amenable to integration into pedagogical curricula, affording students valuable hands-on experiences in piezoelectric control, soldering techniques, and equipment debugging.
• Within the research realm, this microscope can provide high-magnification qualitative imaging of sample surfaces and the capability to perform tunneling bias voltage curve tests.

## Design files summary

**Table:** 

Design file name	File type	Open source license	Location of the file

3DModels	STEP, PDF, SLDPRT, SLDASM files	CC-BY-SA-4.0	https://doi.org/10.17632/f35c6xzzcm.1
HardwareCode	BIN, ELF, and VS code project files	CC-BY-SA-4.0	https://doi.org/10.17632/f35c6xzzcm.1
PCB	ZIP, PDF, and Easy EDA project files	CC-BY-SA-4.0	https://doi.org/10.17632/f35c6xzzcm.1
PythonScript	Python files	GPLv3	https://doi.org/10.17632/f35c6xzzcm.1

### 3D Models

The STM body is predominantly crafted through Computer Numerical Control (CNC) machining of aluminum blocks, necessitating the machining of a total of seven components. The original Solidworks design and STEP files for the manufacturing process are provided within this designated folder. Drill information is documented in the corresponding PDF files.

### PCB

The STM configuration incorporates eight Printed Circuit Boards (PCBs), three dedicated to the control unit, one for the pre-amplifier, and four for circuitry interconnection. These PCBs were designed utilizing EasyEDA (or JLC EDA in China), and the folder includes project files, schematics, and Gerber files requisite for PCB manufacturing.

### HardwareCode

The central controller for the circuit control section employs the ESP32-WROOM-32E-N8 microcontroller module. This folder contains the microcontroller firmware and source code (written using the ESP-IDF framework with PlatformIO).

### PythonScript

The operational software, crafted in Python, is contained within this folder. The necessary libraries (with specific versions) should be installed, and the software can be initiated either by executing the provided “launch.bat” file or through the command line using “python main.py.”.

## Bill of materials summary

**Table:** 

Designator	Component	Number	Cost per unit - CNY	Total cost - CNY	Source of materials	Material type
Control Board	See PCB/BOM_ControlBoard.xlsx	1	787.46	787.46	LCSC and Mouser	Semiconductor
MCU Board	See PCB/BOM_MCUBoard.xlsx	1	32.28	32.28	LCSC	Semiconductor
Power Board	See PCB/BOM_PowerBoard.xlsx	1	110.82	110.82	LCSC	Semiconductor
Pre-amplifier	See PCB/BOM_PreAmp.xlsx	1	237.61	237.61	LCSC and Mouser	Semiconductor
Piezo stack	AL1.65 × 1.65 × 5D-4F	1	32.26	32.26	Taobao	Ceramic
Linear slider	BSP715	1	204	204	Taobao	Metal
Base	STM CNC Block part1	1	48.84	48.84	Sanweihou	Metal
Preamp_cover	STM CNC Block part2	1	35.82	35.82	Sanweihou	Metal
Scanner_cover	STM CNC Block part3	1	40.84	40.84	Sanweihou	Metal
Sample_Table	STM CNC Block part4	1	25.80	25.80	Sanweihou	Metal
PZM_Base	STM CNC Block part5	1	54.01	54.01	Sanweihou	Metal
Scanner_Mount	STM CNC Block part6	1	43.51	43.51	Sanweihou	Metal
Shell	STM 3D print component part1	1	17.23	17.23	Sanweihou	Polymer
Shell_cover	STM 3D print component part1	1	11.83	11.83	Sanweihou	Polymer
Magnent(square)	E-ACTD-W10-H2-T2	2	1.31	2.62	JLCFA	Metal
Magnent(round)	ACTA-B2-D3-L2	1	1.28	12.8	JLCFA	Metal
Piezo Buzzer	7BB-12–9	1	2.19	2.19	Mouser	Ceramic
Pt Wire	Tip(D = 0.3 cm, L = 1 cm)	1	7.2	7.2	ARITER	Mental
PCBs	Control board, MCU board, power board, pre-amplifier board, and boards used for interfacing	8	20	160	JLCPCB	Inorganic
MMCX Connector	KH-MMCX-KE-STM	1	3.52	3.52	LCSC	Metal
IPEX Connector	U.FL-R-SMT-1(10)	9	1	9	LCSC	Metal
FPC Connector	THD0510-05CL-GF	1	0.5	0.5	LCSC	Polymer
FPC Cable	JS05A-05P-030–3-4	1	0.5	0.5	LCSC	Polymer
Coaxial cable A	SMA to IPEX(40 cm)	6	14.8	88.8	XINQY	Polymer
Coaxial cable B	DOSIN-811-0211A(SMA to SMA)	3	18.08	52.24	LCSC	Polymer
IDC Cable	2 × 7Pin, 10 cm	1	1.05	1.05	Taobao	Polymer
M3 × 6 Screw	EDLA-J2-M3-L6	6	0.11	0.66	JLCFA	Metal
M2 × 4 Screw	EDLA-J2-M2-L4	7	0.16	1.12	JLCFA	Metal
M2 × 3 Screw	EDLA-J2-M2-L3	2	0.26	0.52	JLCFA	Metal
M3 × 10 Screw	EDLA-J2-M3-*L*10	4	0.1	0.4	JLCFA	Metal
M5 × 6 Screw	EDLA-J2-M5-L6	4	0.12	0.48	JLCFA	Metal
Hexagon Standoffs	EJLC-M3-L16	12	0.5	6	JLCFA	Metal

## Build instructions

### Damping system set-up

For constructing the damping system, methods including but not limited to suspension and stacked metal plates with Viton can be employed. This article does not provide a specific method, but we offer our construction approach for referenc.

**Fig. 4:**
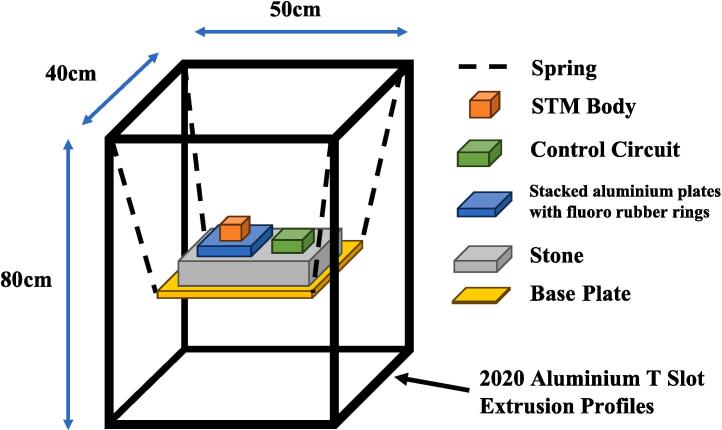
Damping system setup.

The configuration of the damping system is delineated in Fig. 4. Our methodology involved the utilization of a rectangular metal frame fabricated from aluminum profiles. Within this framework, a 10 kg stone was suspended through the integration of four springs, each characterized by a spring length (l) of 20 cm, a spring constant (k) of 408 N/m, and gravitational acceleration (g) set at 9.8 m/s^2^ for each spring. Additionally, a stacking arrangement comprising three aluminum plates (20 × 20 × 1 cm) overlaid with fluoro rubber rings was positioned atop the suspended stone to enhance the damping effect further. The STM body was situated on the stack, while the control circuit was positioned on the stone.

### Control circuit construction

In this step, we will utilize the files from the PCB folder.

#### PCB fabrication

Primarily, the initial step involves accomplishing the fabrication of the PCBs by supplying all the PCB manufacturing files from the PCB/Gerber folder to the PCB manufacturer. The ensuing table delineates the specific process parameters employed during the fabrication of the eight PCBs.

#### PCB assembly

**Fig. 5:**
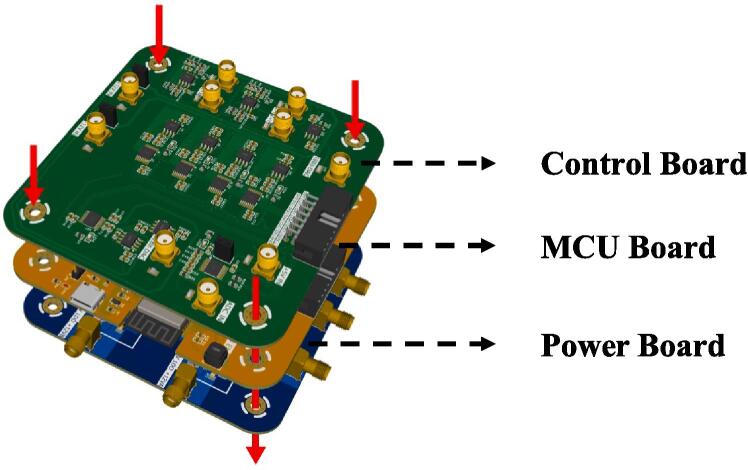
Installation diagram for the control unit. Secure them using hexagonal standoffs as indicated by the red arrows, paying attention to the stacking order. (For interpretation of the references to colour in this figure legend, the reader is referred to the web version of this article.)

**Fig. 6:**
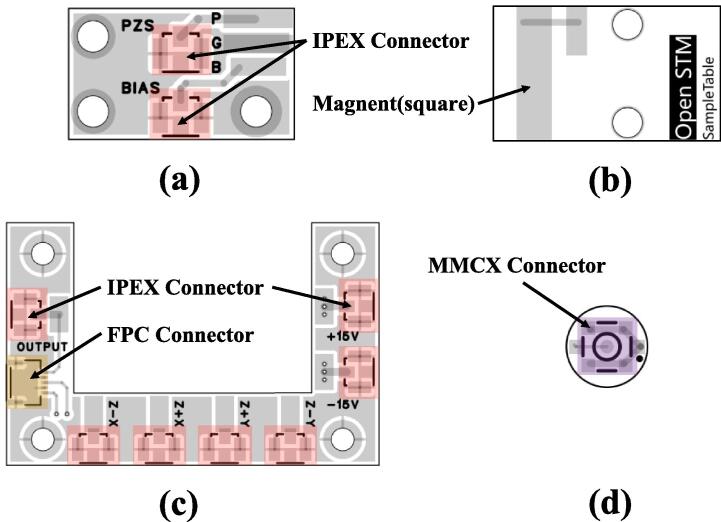
Soldering Diagram. **(a)** Sample_ConnectBoard, the IPEX connectors in figures (a) and (c) are of the same model. **(b)** SampleTable, when soldering the square magnet, consider using low-temperature solder **(c)** ScannerConnector **(d)** ScannerMount.

Kindly consult the respective Bill of Materials (BOM) tables corresponding to the **Control Board**, **MCU Board**, **PreAmp**, and **Power Board** for the soldering procedures. The **Control Board**, **MCU Board**, and **Power Board** collectively constitute the control unit of the STM and necessitate sequential securing using hexagonal standoffs, as illustrated in Fig. 5. The remaining PCBs designated for interfacing purposes should be appropriately soldered, as indicated in Fig. 6, these PCBs are to be affixed to the STM body. Subsequent sections will elucidate the installation methods and delineate the electrical connections among the various PCBs.

#### Control circuit diagram

**Fig. 7:**
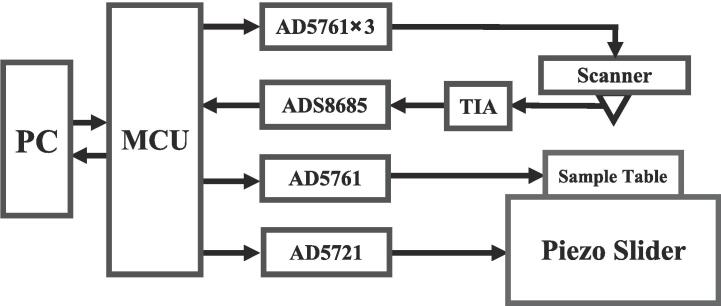
Block diagram of the control system.

Within the context of STM, the customary resolution for digital-to-analog and analog-to-digital converters (DAC and ADC) is typically set at 12 bits or higher [16], [17], [18]. Following a comprehensive evaluation of the trade-off between resolution and cost considerations, our implementation incorporates four 16-bit DACs (AD5761) to regulate the scanner and apply bias to the sample, in conjunction with one 16-bit ADC (ADS8685) tasked with detecting the output signal from the pre-amplifier. Additionally, one 12-bit DAC (AD5721) is employed to control the piezoelectric slider, as depicted in Fig. 7. For the amplification of the tunneling current, a straightforward *trans*-impedance amplifier (TIA) was adopted, utilizing an operational amplifier, specifically the OPA627, for its typical input bias current of 1 pA [19]. This amplifier is adept at detecting and amplifying the tunneling current with a feedback resistor of 100 MΩ.

### STM body assemble

The STM body encompasses four integral components: the scanner block, sample table, piezoelectric slider, and base block. To proceed with the construction, preparation of all STM CNC Blocks (designated as parts 1 to 6) is imperative. The requisite machining files are accessible within the 3DModels/CNC folder. Employing 6061 aluminum alloy or equivalent metal material for the machining process is recommended, with particular attention to maintaining tolerances within the range of ± 0.1 mm. Follow the corresponding PDF drawings for tapping as well.

#### Scanner

**Fig. 8:**
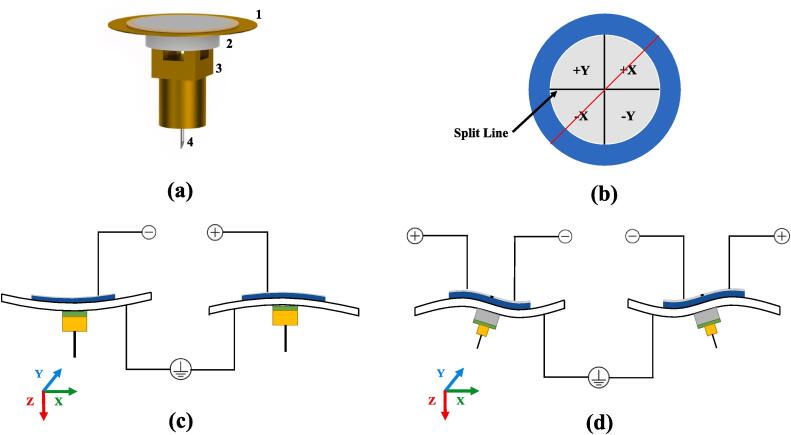
Composition and operation principle of the scanner. **(a)** Composition of different parts of the scanner.1: Piezoelectric buzzer. 2:ScannerMount PCB. 3: MMCX connector. 4:Tip(Pt wire) **(b)** Illustration of buzzer segmenting. **(c)** Movement of the scanner along the Z-Axis. **(d)** Movement of the scanner along the X/Y-Axis.

In this procedural step, the preparation of components, namely the piezoelectric buzzer (7BB-12–9), **ScannerMount** (PCB), and an MMCX connector, is imperative, as depicted in Fig. 8(a). The piezoelectric buzzer typically comprises three layers: a silver-plated layer, a piezoelectric ceramic layer, and a metal layer. The following steps outline the preparatory measures:

(1) **Division of Silver-Plated Layer:** To facilitate the three-dimensional movement of the scanner, the top silver-plated layer of the buzzer must be meticulously divided into four quadrants, employing a cutter knife or equivalent tools, as illustrated in Fig. 8 (b).
(2) **Soldering of MMCX Connector:** Subsequently, the MMCX connector is soldered onto the **ScannerMount** PCB.
(3) **Connection of Enamel-Coated Wires:** Post-soldering of the connector, enamel-coated wires with a diameter of 0.1 mm and a length of approximately 5 cm are soldered to two PCB pads adjacent to the MMCX connector. One wire links to the MMCX connector shell (marked with a dot on the PCB), while the other connects to the MMCX signal end.
(4) **Attachment of PCB and Buzzer:** Employing adhesive glue (Egro 5400 in this instance), affix the PCB and the buzzer, ensuring concentric alignment.
(5) **Soldering of Electrodes:** Conclusively, employ low-temperature solder and enamel-coated wires to solder electrodes onto the four regions of the buzzer.

During the operation of the scanner, the buzzer's metal layer is set to 0 V (ground). The application of equal positive or negative voltages to the four silver-plated layers (+X/-X/+Y/-Y, as delineated in Fig. 8 (b)) induces movement along the Z-axis (illustrated in Fig. 8 (c)). When observing from the side along the direction indicated by the red line in Fig. 8 (b), the impact of applying different voltages to + X/-X becomes evident: utilizing a positive voltage to + X and a negative voltage to -X prompts the scanning head to move rightward, and conversely, for leftward movement. The same principle applies to + Y/-Y. By modulating the voltage across the + X/-X/+Y/-Y quadrants, the three-dimensional movement of the scanner can be accomplished.

#### Scanner Block

For the assembly of the scanner block, preparation of the following components is requisite: **ScannerConnector**(PCB), **Preamp_cover** (STM CNC Block part2), **Scanner_cover** (STM CNC Block part3), **Scanner_Mount** (STM CNC Block part6), Scanner (already prepared in the previous step), Pre-amplifier, M3 × 6 screws(8pcs), and FPC cables.

**Fig. 9:**
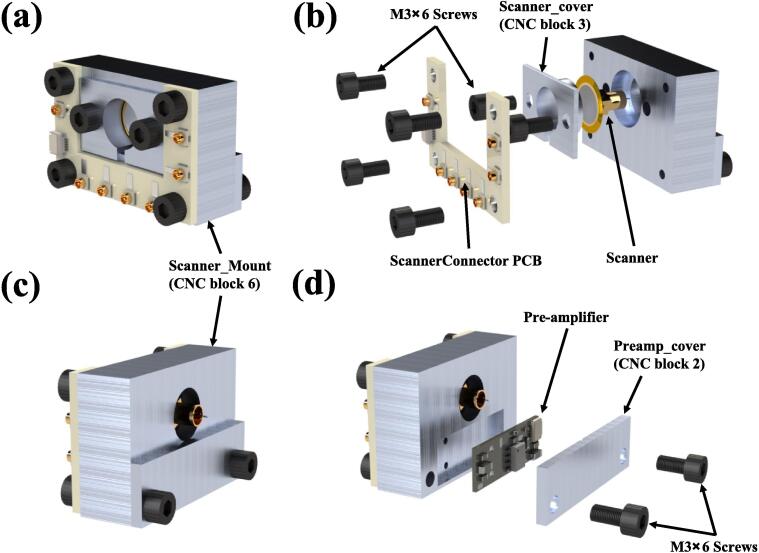
Assembly diagram of the Scanner Block. (a) Front view of the Scanner Block. (b) Explosion front view of the Scanner Block, shows the need to secure the **ScannerConnector** PCB and scanner. (c) Rear view of the Scanner Block. (d) Explosion rear view of the Scanner Block, indicating the need to secure the Pre-amplifier in the **Scanner_Mount**.

Assemble the front half of the block as follows: (1)As shown in Fig. 9(b), place the **Scanner_cover** over the scanner and install them into the block, securing it with screws. (2)Use screws to attach the **ScannerConnector** PCB to the block. (3) Solder the electrodes of the scanner onto the corresponding pads on the **ScannerConnector** PCB. Please refer to Fig. 8(b) and the silkscreen markings on the PCB for the correct sequence.

**Fig. 10:**
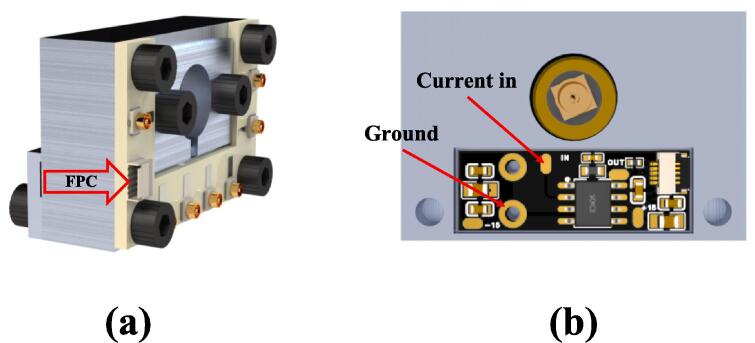
Wires to be installed on the pre-amplifier. (a) Connected to the ScannerConnector PCB through the FPC cable. (b) The enamel-coated wires from the scanner need to be soldered onto the two indicated solder pads.

For the rear half of the block assembly, use the **Preamp_cover** to mount the pre-amplifier(**PreAmp**) inside the block and secure it with screws. However, please note that before covering the pre-amplifier with the **Preamp_cover**, it is necessary to insert the FPC cable into the pre-amplifier and connect the other end of the cable, as shown in Fig. 10(a), to the **ScannerConnector** PCB. Additionally, the enamel-coated wires were soldered from the scanner to their respective positions on the pre-amplifier, as shown in Fig. 10(b). The MMCX connector's shell should be connected to the ground for electromagnetic shielding, and the signal end should be connected to “Current in” to conduct tunneling current.

#### Piezoelectric slider and sample table

**Fig. 11:**
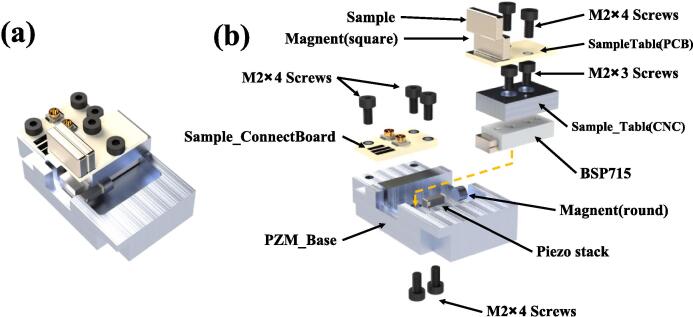
Assembly diagram of the piezo slider and sample table. (a) Side view (b) Exploded view.

Preceding the assembly process, it is imperative to prepare the ensuing components: **Sample Table**(PCB), **Sample_Table**(CNC Block part 4), BSP 715 linear slider, magnet (round), magnet (square), piezo stack (AL1.65 × 1.65 × 5D-4F), **Sample_ConnectBoard** (PCB), **PZM_Base** (CNC Block part 5), M2 × 4 screws (7 pcs), M2 × 3 screws (2 pcs). Subsequently, embark on the assembly procedure by adhering to the guidelines presented in Fig. 11:

(1) Secure the **Sample_ConnectBoard**(PCB) on the **PZM_Base**(CNC Block) using screws.
(2) Use screws to fasten the BSP715 linear slider onto the **PZM_Base**(CNC Block). During this step, ensure the slider is parallel to the installation groove.
(3) Attach the piezo stack to the edge of the **PZM_Base**(CNC Block) using adhesive, as shown in Fig. 11(a) and Fig. 11(b). Then, solder the two electrodes of the piezo stack to the **Sample_ConnectBoard**(PCB), with the red wire connected to G(G means Ground) and the orange wire connected to P(P means positive. However, the voltage output from here will be bipolar).
(4) Affix the magnet(round) to the slider (note: attach the magnet on the side, not on the bottom or top). Push the slider to make contact between the magnet and the piezo stack. Then, use adhesive to secure the magnet to the piezo stack. At this point, the piezo slider assembly is complete.
(5) Secure the **Sample_Table**(CNC Block) onto the slider using screws. It is essential to acknowledge that, owing to tolerances, the screws may exceed the requisite length. In the event of such an occurrence, a viable solution is to employ adhesive on the screw heads, serving as spacers upon solidification.
(6) Fix the **SampleTable**(PCB) onto the **Sample_Table**(CNC), and use enamel-coated wire to connect the smaller square solder pad on the **SampleTable**(CNC) to pad B(B means Bias) on the **Sample_ConnectBoard**(PCB).

#### Assemble

**Fig. 12:**
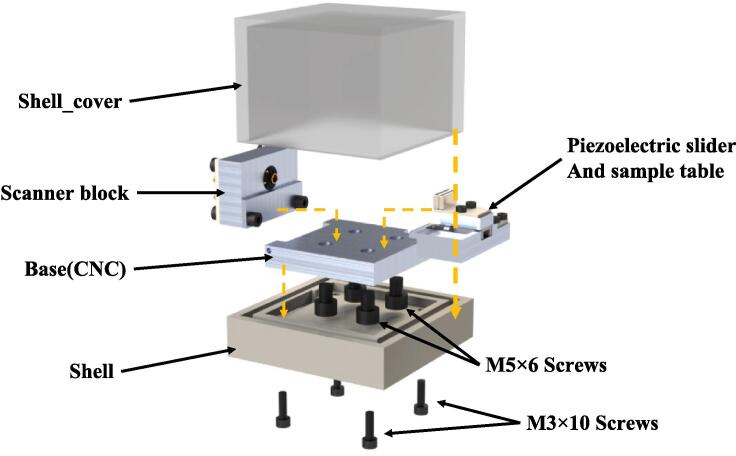
Schematic of the leading microscope assembly. The **shell** and **shell_cover** together form the dust cover.

Conduct the ultimate assembly by the delineated sequence illustrated in Fig. 12. It is noteworthy that when employing M5 screws to affix the piezoelectric slider, caution should be exercised to avoid overtightening, as this could potentially modify the magnetic force acting on the BSP715. While the inclusion of the dust cover is discretionary, its removal can result in a further reduction in the microscope's dimensions. However, it is essential to recognize that the dust cover serves the purpose of preventing dust intrusion and mitigating disturbances caused by airflow. Table 1

**Table 1:** Parameters used in PCB manufacturing.

PCB file name	Material	Board Thickness(mm)
Gerber_ControlBoard	FR-4	1.6
Gerber_MCUBoard	FR-4	1.6
Gerber_PowerBoard	FR-4	1.6
Gerber_PreAmp	FR-4	1
Gerber_Sample_ConnectBoard	FR-4	1.6
Gerber_SampleTable	FR-4	1.6
Gerber_ScannerConnector	FR-4	1.6
Gerber_ScannerMount	FR-4	1.6

### Wiring

Please refer to Table. 2 to establish connections between PCBs using different types of coaxial cables. The coaxial cables should pass through the wire groove on the shell when connecting the control unit to the STM body.

**Table 2:** Wiring table.

Source	Target	Source Silkscreen	Target Side Silkscreen	Function Describe	Wire type
Power Board	Control Board	V_LDO_-12VA	−12V_IN	DAC Power	SMA-SMA
Power Board	Control Board	V_LDO_-12VB	+12V_IN		SMA-SMA
Power Board	Scanner_Connector	V_LDO_+12VA	+12 V	Pre-amplifier power	SMA- IPEX
Power Board	Scanner_Connector	V_LDO_+12VB	−12 V		SMA- IPEX
Power Board	Control Board	V_LDO_+5V	+5V_IN	ADC Power	SMA-SMA
MCU Board	Power Board	PWR_OUT	BOOSTER_IN	5 V Power	SMA-SMA
Control Board	Scanner_Connector	Z-Y	Z-Y	Scanner control	SMA- IPEX
Control Board	Scanner_Connector	Z + Y	Z + Y		SMA- IPEX
Control Board	Scanner_Connector	Z-X	Z-X		SMA- IPEX
Control Board	Scanner_Connector	Z + X	Z + X		SMA- IPEX
Control Board	Sample_ConnectBoard	16Bit-DAC	BIAS	Sample bias voltage	SMA- IPEX
Control Board	Sample_ConnectBoard	12Bit-DAC	PZS	Piezoelectric slider driver	SMA- IPEX
Control Board	Scanner_Connector	ADC_IN	OUTPUT	Pre-amplifier output	SMA- IPEX

### Tip and sample preparation

In an idealized context, an STM tip typically comprises only a few atoms. The established protocol for tip preparation involves electrochemical etching utilizing a tungsten wire [20]. While this method can yield a tip with noteworthy sharpness, the preparation process is both time-consuming and intricate. Alternatively, in practical applications, the utilization of platinum-iridium alloy wire followed by cutting can also generate tips that meet the stringent requirements of an STM [21]. Platinum, known for its inert characteristics, effectively mitigates oxidation post-cutting, and the alloy of platinum and iridium further fortifies the tip's hardness. In an endeavor to streamline the tip manufacturing process, an experiment was conducted using pure platinum wire, yielding successful outcomes. The fabrication method entails swabbing a 0.3 mm diameter platinum wire with alcohol, securing one end of the platinum wire with tweezers, and stretching and cutting the other end at an angle of approximately 45 degrees using wire cutters. Subsequently, the cut tip is delicately inserted into the MMCX connector of the scanner.

The fabrication of the sample involves the utilization of a magnet(square). The procedural steps are delineated as follows: Initially, affix the test material onto the magnet using adhesive. Subsequently, establish an electrical connection between the sample and the magnet by applying conductive silver paste. Upon drying the conductive silver paste, secure the prepared sample to the sample table through magnetic adsorption, and then the scanning can proceed.

## Operation instructions

This section elucidates the preliminary procedures preceding microscope imaging, including the software operations and adjustments to parameters for the piezo stage and approach algorithm.

### Hardware setting up

(1) Use jumper caps to connect the **Control Board** jumpers to power the DACs and ADCs: H1, H2, and H3.
(2) Use jumper caps to connect all jumpers named PWR on the **Power Board**, connect the SS and V_EN jumper, connect COMP1 and COMP2 to the GND terminal, SYNC/FREQ to the 2.4 M terminal, SEQ to the SEQ. Terminal, SLEW to the NORM terminal, EN1 to the ON terminal, and EN2 to the OFF terminal.
(3) Connect the jumpers PWR_DEBUG_EN and USB_EN on the **MCU Board**.
(4) Connect a low-ripple USB 5 V Type-C power supply to the USB-C PWR port. Connect the USB on the computer to the USB_DEBUG port.
(5) Use tweezers to insert the tip into the scanner.
(6) Load the sample.
(7) Move the piezoelectric slider to bring the sample close to the tip, about 1 mm.
(8) Cover it with the dust cover and complete the scanning on the software side.

### Software interface

The software interface comprises five sections: UART Connect box, Slider Approach Control box, Tunneling Current Monitoring box, and a multi-page testing tab. A typical testing process includes the following steps:

(1) Click Refresh in the UART Connect box to select the corresponding serial port.
(2) Click the approach button in the Approach box and wait for the approach to complete.
(3) In the multi-page Testing Tab, select the appropriate testing category, adjust parameters, and complete the test.
(4) Save the corresponding file(Curve data or image).

Curve data is saved as an Excel file, while images are saved as TIFF files. Image processing can be performed using other software, such as Gwiddion.

### Piezoelectric slider setting

Liao et al. elucidated the operational principle of the positioner founded on the stick–slip phenomenon [8]. The functionality of the positioner manifests in two modes: the high-resolution mode, where a triangular wave is applied to the piezo stack, and the low-resolution mode, which employs a sawtooth wave for the piezo stack. Given that high resolution is unnecessary during the pre-approach phase and considering the imperative to economize time, our slider exclusively operates within the low-resolution mode.

**Fig. 13:**
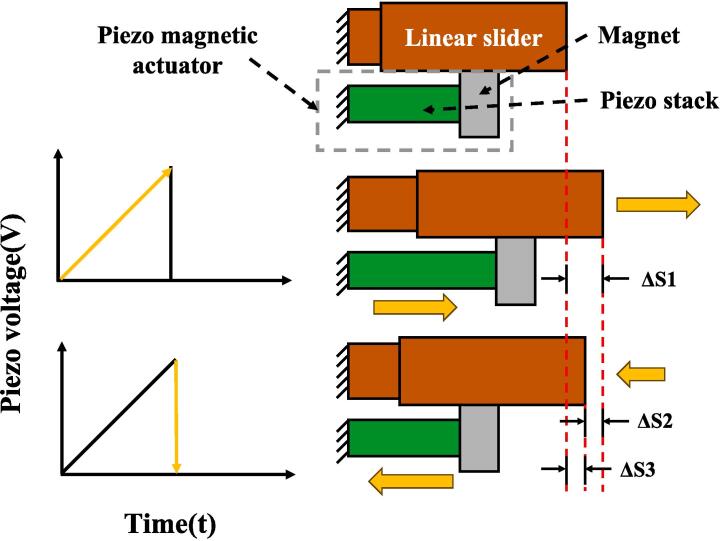
Piezoelectric slider working diagram in low-resolution mode.

Fig. 13 elucidates the operational mechanism of the piezoelectric slider. The extension of the piezo stack, induced by an elevation in piezo voltage, impels the slider to shift to the right by ΔS1, influenced by the magnetic force. Then, the piezo stack swiftly contracts when the piezo voltage reverts to zero. Owing to the stick–slip phenomenon [22], the slider fails to entirely synchronize with the piezo stack's movement, resulting in a displacement of ΔS3. Throughout this progression, ΔS1 signifies the displacement during synchronous movement of the slider with the piezo stack, ΔS2 denotes the displacement engendered by the piezo stack's contraction propelling the slider, and ΔS1 = ΔS2 + ΔS3.

In the software settings page, the Slider Amplitude parameter can be modified to control ΔS1 of the slider. A larger ΔS1 will result in a faster speed but may increase the risk of the tip crashing.

### Approach method

**Fig. 14:**
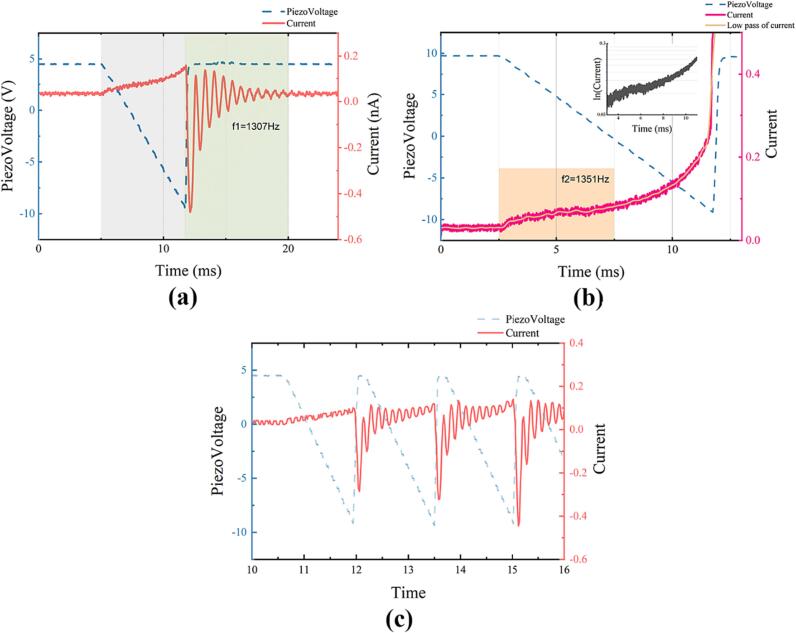
Pre-amplifier (TIA) output signal vs piezoelectric slider voltage, sample bias voltage |V_bias_|=10 V, Highly Oriented Pyrolytic Graphite (HOPG) sample from JH Special Carbon Technology Co., LTD, the curve is sampled using a RIGOL 1102Z-E oscilloscope. (a) Response of the TIA to a single-step movement of the slider.(b) Response of the TIA to a single-step slider movement, with tip collision occurring after this step. (c) Response of the TIA to continuous multiple-step movements of the tip.

During the movement of the piezoelectric slider, a discernible fluctuation occurs in the output signal of the pre-amplifier when the tip approaches the sample sufficiently. Fig. 14(a) provides a clear illustration of this phenomenon. The blue dashed line in the figure signifies the piezo stack driving voltage, wherein negative voltage propels the sample toward the tip. The pre-amplifier registers a positive current in the gray region as the distance between the tip and the sample diminishes. In the green area, during the phase of relative sliding between the piezo stack and the slider, the current abruptly reverses and displays oscillation. To further elucidate the source of this current, we allowed the slider to continue its movement and captured the curve change just before the tip collision, as depicted in Fig. 14 (b). By applying a logarithmic transformation to the curve, we identified that the current exhibits exponential changes, indicating the dominance of the tunneling current in the gray region.

The occurrence of polarity reversal in the oscillation region excludes the possibilities of breakdown, electron emission, and tunneling current [23]. Given that the capacitance between the tip and the sample can yield a detectable signal [15], [24], we posit that the current oscillation in this segment arises from brake moan oscillation induced by the friction between the magnet on the piezo stack and the slider [25]. The brake moan oscillation between the tip and sample leads to a capacitance change, given the constant bias voltage, consequently causing a current oscillation. In fact, in Fig. 14 (b), this oscillation can also be observed, and its frequency is close to that in Fig. 14 (a). This oscillation intensifies as the distance between the sample and the tip diminishes. Leveraging this signal as a characteristic value, we estimate the sample-tip distance and enhance the efficiency of the pre-approach procedure. Fig. 14 (c) illustrates the current variation during continuous slider movement, revealing a notable increase in oscillation amplitude as the slider advances.

The entire approach is delineated into three stages: fast mode, slow mode, and Z scan mode. During the fast mode, the slider moves at a predetermined speed, detecting the peak of current oscillation after each step. Upon reaching a specified threshold (In software settings, Fast Cap Current), the system transitions into slow mode. In slow mode, the slider's movement speed decelerates. The system then advances to Z Scan mode when the oscillation peak attains a second predetermined threshold(In software settings, Slow Cap Current). Within Z Scan Mode, the system controls the scanner in the Z-direction with each step to ascertain whether the sample has entered the scanner's scanning range. The approach concludes if the tunneling current attains the set point during this process. All the threshold parameters mentioned above are configurable in the settings tab. With the parameters appropriately configured, the sample loading process takes approximately 1 min to establish the tunneling current.

## Validation and characterization

We utilized a 0.5 mm thick Highly Oriented Pyrolytic Graphite (HOPG) sample sourced from JH Special Carbon Technology Co., LTD for our experimental investigations. The tests were conducted employing the described vibration isolation system within an office environment situated on the 4th floor.

**Fig. 15:**
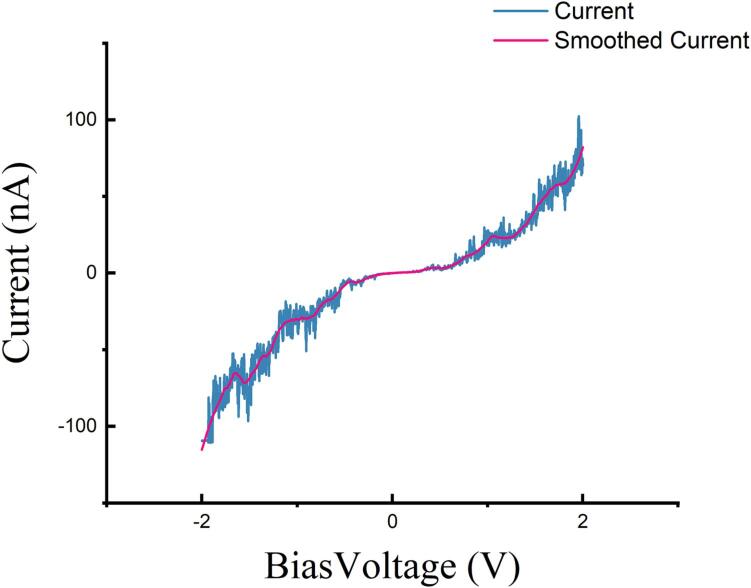
Bias voltage vs tunneling current of HOPG, range from −2V to 2 V.

Fig. 15 illustrates the outcomes of our tests on the bias curve of HOPG. Once the sample and tip reached the tunneling distance, we systematically varied the sample bias voltage across the range of −2V to 2 V while recording the resulting current output. Upon comparative analysis with the curves obtained by other researchers [26], [27], our measured curve exhibits similarities in characteristics.

**Fig. 16:**
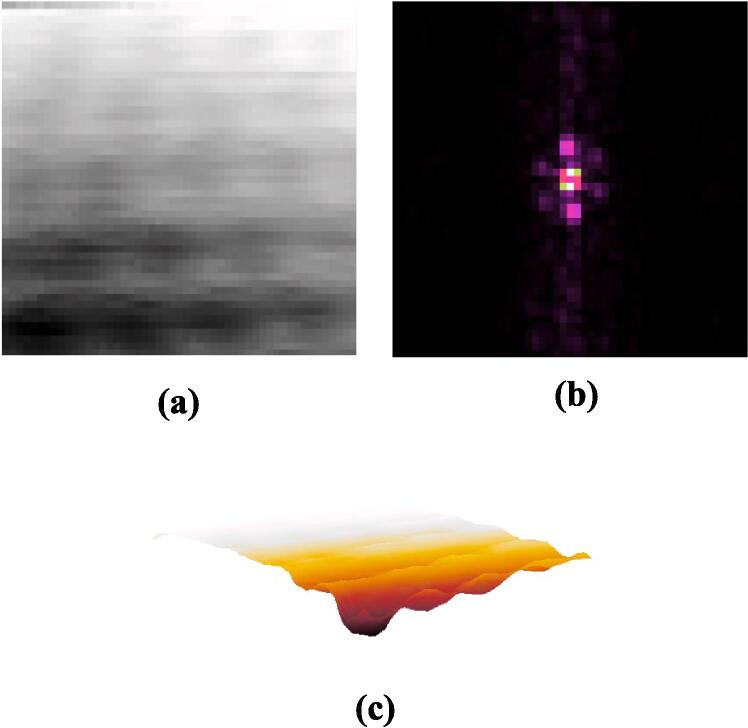
Raw scanning tunneling microscope image of HOPG, 50 × 50 sample area, X/Y scan voltage range from 0 ∼ 15.25 mV,|V_bias_|=80 mV, scan time = 657 ms. (a) 2D gray level image (b) FFT of raw image (c) 3D view of the image.

Subsequently, we conducted STM imaging of the HOPG sample. Fig. 16(a) portrays the raw image of HOPG, acquired with a scan size of 50 × 50, a sample bias voltage of 80 mV, and a scan time of 657 ms. The triangular structure of graphite atoms is discernible in the STM image pattern from Fig. 16 (a). Fig. 16 (b) illustrates the outcome following a two-dimensional fast Fourier transform applied to the raw image, dominated by six Fourier coefficients at an angle of approximately 120°. This aligns with the anticipated behavior of HOPG under scanning tunneling microscopy [28].

**Fig. 17:**
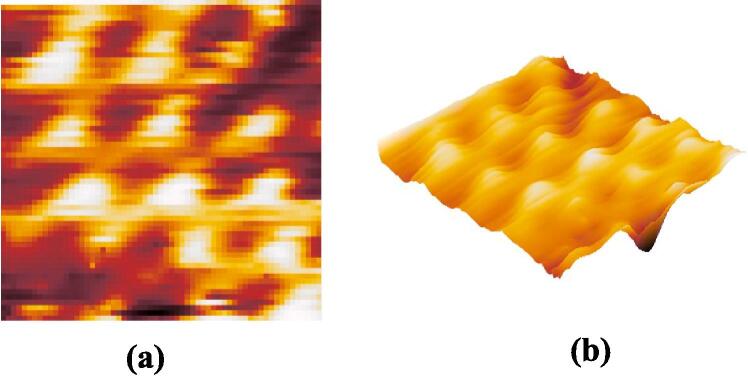
Scanning tunneling microscope image of HOPG, 60 × 60 sample area, X/Y scan voltage range from 0 ∼ 18.3 mV,|Vbias|=50 mV. (a) Proceed HOPG image with Gwiddion (b) 3D view of HOPG image.

Various scans of the same HOPG sample were conducted by altering the bias voltage, adjusting the number of sampling points, and interchanging the tip. All scans consistently revealed the atomic characteristics of HOPG. Fig. 17(a) showcases the result obtained at a sample bias voltage of 50 mV and a scan size of 60 × 60, presenting a more pronounced lattice structure.

## Declaration of competing interest

The authors declare that they have no known competing financial interests or personal relationships that could have appeared to influence the work reported in this paper.
